# THE ASSOCIATION BETWEEN MEDICAL MARIJUANA USE AND DIVIDED ATTENTION ON A SIMULATED DRIVING PERFORMANCE TASK

**DOI:** 10.1093/geroni/igad104.0035

**Published:** 2023-12-21

**Authors:** Nicole Ennis, Daniel Dunleavy, Katie Kloss, Nichole Stetten, Sherrilene Classen

**Affiliations:** Florida State University, Tallahassee, Florida, United States; Florida State University-College of Medicine, Tallahassee, Florida, United States; Florida State University-College of Medicine, Tallahassee, Florida, United States; University of Florida, Gainesville, Florida, United States; University of Florida, Gainesville, Florida, United States

## Abstract

Driving is a complex and dynamic task requiring divided attention (DA), awareness of the traffic and surroundings while focusing on critical stimulus. The goal of this project was to examine the relationship between medical marijuana use and divided attention in adults 50 and older during a simulated driving performance task. We recruited adults 50 and older (N=20) pre-exposure to medical marijuana and followed them for the first 3 months of use. Driving performance was examined at baseline (pre-exposure to medical marijuana) and 1-month post medical marijuana exposure During a driving performance identification task that appeared in central or peripheral vision during the simulated drive, DA was assessed as a triangle that appeared on screen at pre-programmed intervals to which participants were required to press a button on the dashboard in response. Results showed that at 1-month post-medical marijuana initiation the 7-day pattern of use could be classified as light, moderate, or heavy. Further, there was a trend of improved performance among medical marijuana users 50 and older with moderate to heavy use having the best performance on the DA. We attribute these findings to the stimulant properties of medical marijuana and improved sleep reported by participants.

